# Whole-exome sequencing analysis identifies distinct mutational profile and novel prognostic biomarkers in primary gastrointestinal diffuse large B-cell lymphoma

**DOI:** 10.1186/s40164-022-00325-7

**Published:** 2022-10-15

**Authors:** Shan-Shan Li, Xiao-Hui Zhai, Hai-Ling Liu, Ting-Zhi Liu, Tai-Yuan Cao, Dong-Mei Chen, Le-Xin Xiao, Xiao-Qin Gan, Ke Cheng, Wan-Jia Hong, Yan Huang, Yi-Fan Lian, Jian Xiao

**Affiliations:** 1 Department of Medical Oncology, the Sixth Affiliated Hospital of Sun Yat-Sen University, Guangzhou, 510655 China; 2 Guangdong Provincial Key Laboratory of Liver Disease Research, the Third Affiliated Hospital of Sun Yat-Sen University, Guangzhou, 510630 China; 3 Department of Pathology, the Sixth Affiliated Hospital of Sun Yat-Sen University, Guangzhou, 510655 China; 4 Department of Hematology, the Sixth Affiliated Hospital of Sun Yat-Sen University, Guangzhou, 510655 China; 5 Guangdong Provincial Key Laboratory of Colorectal and Pelvic Floor Diseases, The Sixth Affiliated Hospital, Sun Yat-Sen University, Guangzhou, 510655 China

**Keywords:** Whole-exome sequencing/WES, Diffuse large B-cell lymphoma/DLBCL, Gastrointestinal tract/GI tract, Mutation profile, *IGLL5*, *LRP1B*

## Abstract

**Background:**

Diffuse large B-cell lymphoma (DLBCL) is the most common aggressive non-Hodgkin lymphoma, and about 10% of DLBCL cases primarily occur in the gastrointestinal tract. Previous reports have revealed that primary gastrointestinal-DLBCL (pGI-DLBCL) harbors different genetic mutations from other nodal or extranodal DLBCL. However, the exonic mutation profile of pGI-DLBCL has not been fully addressed.

**Methods:**

We performed whole-exome sequencing of matched tumor tissues and blood samples from 53 pGI-DLBCL patients. The exonic mutation profiles were screened, and the correlations between genetic mutations and clinicopathological characteristics were analyzed.

**Results:**

A total of 6,588 protein-altering events were found and the five most frequent mutated genes in our pGI-DLBCL cohort were *IGLL5* (47%), *TP53* (42%), *BTG2* (28%), *P2RY8* (26%) and *PCLO* (23%). Compared to the common DLBCL, significantly less or absence of *MYD88* (0%), *EZH2* (0%), *BCL2* (2%) or *CD79B* (8%) mutations were identified in pGI-DLBCL. The recurrent potential driver genes were mainly enriched in pathways related to signal transduction, infectious disease and immune regulation. In addition, HBV infection had an impact on the mutational signature in pGI-DLBCL, as positive HBsAg was significantly associated with the *TP53* and *LRP1B* mutations, two established tumor suppressor genes in many human cancers. Moreover, *IGLL5* and *LRP1B* mutations were significantly correlated with patient overall survival and could serve as two novel prognostic biomarkers in pGI-DLBCL.

**Conclusions:**

Our study provides a comprehensive view of the exonic mutation profile of the largest pGI-DLBCL cohort to date. The results could facilitate the clinical development of novel therapeutic and prognostic biomarkers for pGI-DLBCL.

**Supplementary Information:**

The online version contains supplementary material available at 10.1186/s40164-022-00325-7.

## Introduction

The incident rate of non-Hodgkin lymphomas (NHLs) in most contries has considerably increased in recent years [1]. Diffuse large B-cell lymphoma (DLBCL) is the most common subtype of NHLs, accounting for nearly one-third of all lymphoid neoplasm in China annually [2, 3]. Though at least two DLBCL subtypes have been identified by RNA expression profiles, the germinal center B-cell-like (GCB) subtype and the activated B-cell-like (ABC) subtype, DLBCL still represents a clinical heterogenous disease due to its complex and diverse histological characteristics [4, 5]. DLBCL patients often present with an aggressive clinical behavior, but most of them can be cured by the standard regimen based on rituximab plus cyclophosphomide, doxorubicin, vincristine and prednisone (R-CHOP) [6]. The application of next-generation sequencing has helped reveal a deep degree of molecular and genetic heterogeneity in hematological diseases, and confirmed that genetic aberrations contribute to occurrence and progression of DLBCL [7, 8].

DLBCL arises from extranodal organs in about 30% of total cases, and one third of extranodal DLBCL cases occur in the gastrointestinal tract, making it the most common primary extranodal site [9, 10]. Patient prognosis and recurrence risk of extranodal DLBCL vary according to the primary site of origin, which may harbor different genetic mutations clarified by high through-put sequencing studies [11, 12]. Primary gastrointestinal DLBCL (pGI-DLBCL) has a significantly decreased level of *MYD88* and *CD79B* mutations compared to nodal DLBCL and other extranodal DLBCL in immune-privileged sites, such as central nervous system and testis [13, 14]. Meanwhile, genetic mutations of *MYC* or *BCL2* rearrangements could be related to the survival and prognosis of pGI-DLBCL patients [15, 16]. The genetic mutation profiles discovered by more in-depth analysis revealed that pGI-DLBCL may have different modes of pathogenesis and progression from non-gastrointestinal DLBCL. Recently, by analyzing a small group of patients using whole-exome sequencing (WES), a study by Li et al*.* has shed a light on the genetic mutations in pGI-DLBCL [17]. However, comprehensive research focusing on the mutational landscape of pGI-DLBCL, and the correlation between its genetic mutations and clinicopathological features are still rare.

In the present study, we aimed to derive the predictive mutational profile by performing capture-based targeted WES on 53 Chinese pGI-DLBCL patients. The association between clinical characteristics and genetic alterations was also explored. In addition, we tried to identify genetic mutations possibly affecting patient survival and their underlying mechanisms. Our study provided a deeper insight into the genetic features of pGI-DLBCL, which may be helpful to clarify the lymphomagenesis process and develop putative therapeutic and prognostic biomarkers for this disease.

## Materials and methods

### Patient Cohort

Fifty-three patients diagnosed with pGI-DLBCL according to the criteria defined by Lewin et al*.* [18] were recruited in this study. All patients underwent partial gastrectomy or enterectomy plus R-CHOP based therapy in our hospital spanning from January 1, 2011 to July 21, 2021. Forty-six surgical resection specimens, seven biopsy specimens and matched patient peripheral blood mononuclear cells (PBMCs) were used for sequencing study. All specimens were reviewed by two independent hematopathologists (Yan Huang and Hai-Ling Liu) according to the 2017 World Health Organization classification criteria [19]. The corresponding medical records of all patients were reviewed to obtain the clinicopathological information. The study was approved by the institutional review board at the Sixth Affiliated Hospital of Sun Yat-Sen University.

### WES

Tumor DNA was isolated from five 5-μm-thick sections of formalin-fixed paraffin-embedded tumor tissues with a minimum of 70% neoplastic cells using QIAamp FFPE DNA Tissue Kit (Qiagen, USA), and the paired normal control DNA of PBMCs was extracted with DNeasy Tissue and Blood Kit (Qiagen, USA) according to the manufacturer’s instructions. Degradation and contamination were monitored on a 1% agarose gel, and the concentration was measured by using a Qubit® DNA Assay Kit in a Qubit® 2.0 Fluorometer (Life Technologies, USA). Qualified genomic DNA from tumors and matched PBMCs from 53 pGI-DLBCL patients were fragmented by Covaris technology with resultant library fragments of 180–280 bp, and then adapters were ligated to both ends of the fragments. Extracted DNA was then amplified by ligation-mediated PCR (LM-PCR), purified, and hybridized to the Agilent SureSelect Human Exome V6 (Santa Clara, USA) for enrichment, and nonhybridized fragments were then washed out. Both uncaptured and captured LM-PCR products were subjected to real-time PCR to estimate the magnitude of enrichment. Each captured library was then loaded onto the Illumina HiSeq X platform (Hangzhou Jichenjunchuang Medical Laboratory Co., Ltd, Beijing, China). We performed high-throughput sequencing for each captured library independently. Tumor and normal DNA samples were sequenced to an average depth of > 100 × and > 40 × in targeted exonic regions, respectively.

### Genomic analysis

After generating raw data through base calling, paired-end reads were trimmed to remove stretches of low-quality bases (< Q10) and adapters in the sequences. The clean reads were mapped to NCBI Build 37 (hg19) using BWA-0.7.12 *mem* with the default settings. SAMtools-1.2 was used to sort and index all the BAM files; PicardTools-1.119 was used to remove the duplicates; and GATK-3.3–0 was used for InDel realignment and base quality score recalibration. MuTect-1.1.4 and Strelka were used to call somatic SNVs and InDels in the paired normal and tumor samples. Variants identified in the 1,000 Genomes database (https://www.1000genomes.org/) with a frequency > 1% (unless they were in the Catalog of Somatic Mutations in Cancer (COSMIC) database) or in the Exome Aggregation Consortium (http://exac.broadinstitute.org/) with a frequency > 0.1% were discarded from the analysis. Variants with an alternate allele depth < 2 and a frequency < 5% were also excluded. In addition, SNVs and InDels were filtered to remove benign changes predicted by the following predictive software programs, including Polyphen2, MutationTaster, Mutation Assessor, FATHMM, Radial SVM, LR, SIFT, and LRT. ANNOVAR was used to annotate all the somatic mutations after filtering.

### Pathway enrichment analysis

Gene clustering analysis of the driver mutations was performed by Database for Annotation, Visualization and Integrated Discovery (DAVID) online tool (https://david.ncifcrf.gov/) as previously described [20]. Only the Kyoto Encyclopedia of Genes and Genomes (KEGG) pathway enrichment analysis which evaluates the modules at the functional level of the selected genes was executed. Bonferroni *P* value < 0.05 was set as the cut-off criterion and regarded as statistically significant.

### Statistical analysis

Statistical analysis was performed using R version 4.1.2 and GraphPad Prism version 7 (La Jolla, CA, USA). The Mann–Whitney U test and the Spearman rank correlation test were employed to analyze the relationship between the mutated genes and clinicopathological characteristics. Survival analysis was performed using Kaplan–Meier curves and compared using the log-rank test. Comparative test differences were considered significant if the 2-tailed *P* value was < 0.05 otherwise indicated.

## Results

### Clinicopathological characteristics of the pGI-DLBCL patient cohort

The clinicopathological characteristics of the pGI-DLBCL patient cohort were summarized in Table 1 and Additional file 1: Table S1. Of note, we included 53 patients diagnosed with pGI-DLBCL in this study, which consisted of 40 males and 13 females, respectively. Tumors were primarily originated from the stomach of 11 patients, small intestine of 29 patients, or large intestine of 13 patients. Helicobacter pylori (Hp) or hepatitis B virus (HBV) infection was positive in 21 (39.6%) or 11 cases (20.8%), respectively. According to the Hans algorithm, 33 and 20 patients were classified as GCB (62.3%) and non-GCB (37.7%) DLBCL subtypes based on the immunohistochemical features. The cohort included 35 patients in clinical stage I or II, and 18 patients in clinical stage III or IV. By the end of the current study, the follow-up duration of the patients was as long as 128.4 months with 11 dead records.

**Table 1:** Clinicopathological characteristics of 53 pGI-DLBCL patients

Characteristics	Patients	
	n	Percentage
Age, years		
≤ 60	28	52.8%
> 60	25	47.2%
Gender		
Male	40	75.5%
Female	13	24.5%
Origin		
Large Intestine	13	24.5%
Small Intestine	29	54.7%
Stomach	11	20.8%
Han’s Algorithm		
GCB	33	62.3%
non-GCB	20	37.7%
B Symptom		
Yes	14	26.4%
No	39	73.6%
Hp Infection		
Positive	21	39.6%
Negative	32	60.4%
LDH Level		
Elevated	31	58.5%
Normal	22	41.5%
Hypoproteinemia		
Yes	45	84.9%
No	8	15.1%
Anemia		
Yes	52	98.1%
No	1	1.9%
HBsAg		
Positive	11	20.8%
Negative	42	79.2%
ECOG PS		
< 2	43	81.1%
≥ 2	10	18.9%
Lugano Stage		
I-II	35	66.0%
III-IV	18	34.0%
IPI		
0–1	28	52.8%
2–5	25	47.2%
Survival		
Alive	42	79.2%
Dead	11	20.8%

### Exonic mutational profile of pGI-DLBCL

**Fig. 1:**
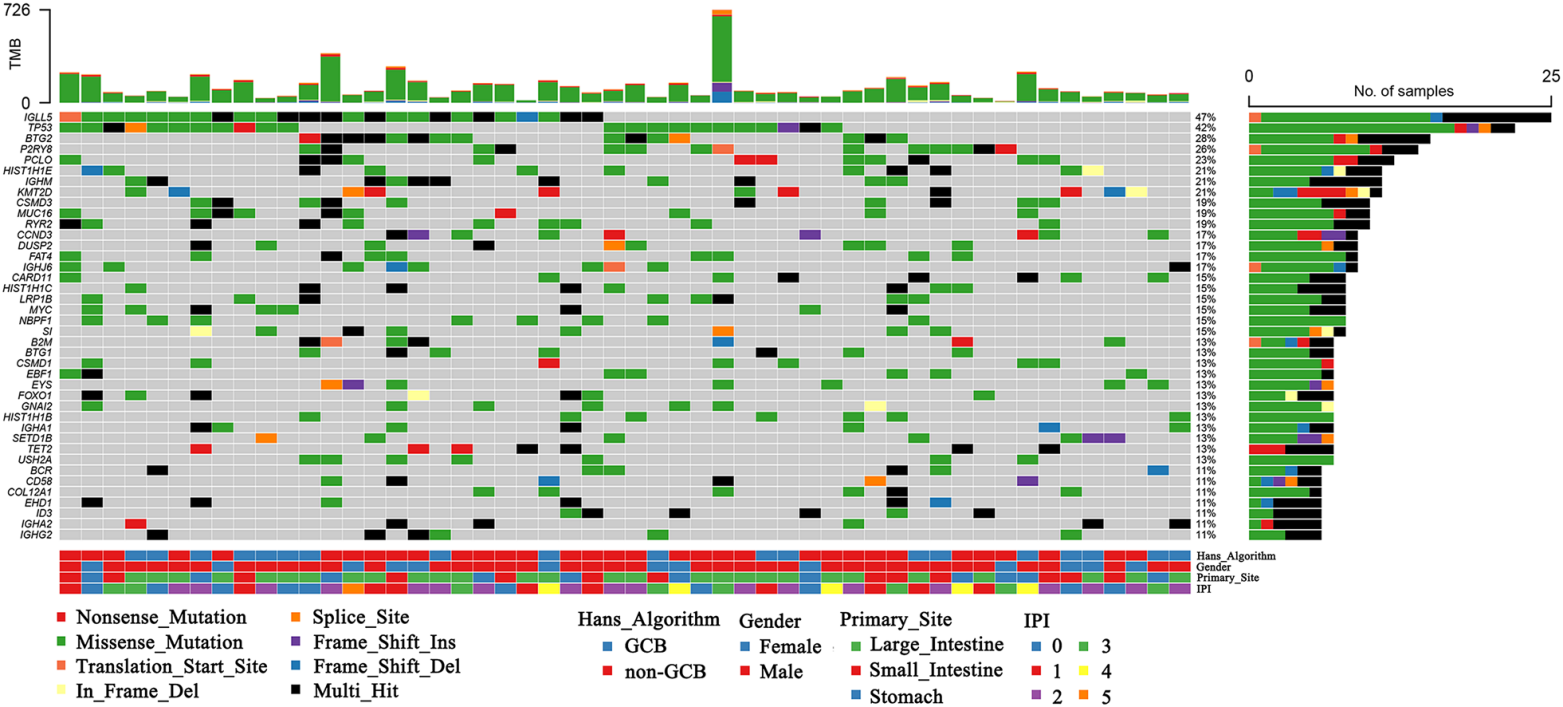
Top 40 mutated genes in 53 pGI-DLBCL patients. The bar graph on the top indicates the absolute number of exonic mutations in each patient. Top 40 frequently mutated genes constitute the individual rows and are arranged by their mutation rates displayed on the right. Each column represents a patient and each row represents a gene. The histogram on the right shows the number of mutations in each gene. The tracks at the bottom provide information on gender, the molecular subtype sorted by Hans algorithm, the primary tumor sites and the IPI that are color-coded as indicated in the legend. TMB: tumor mutational burden

**Fig. 2:**
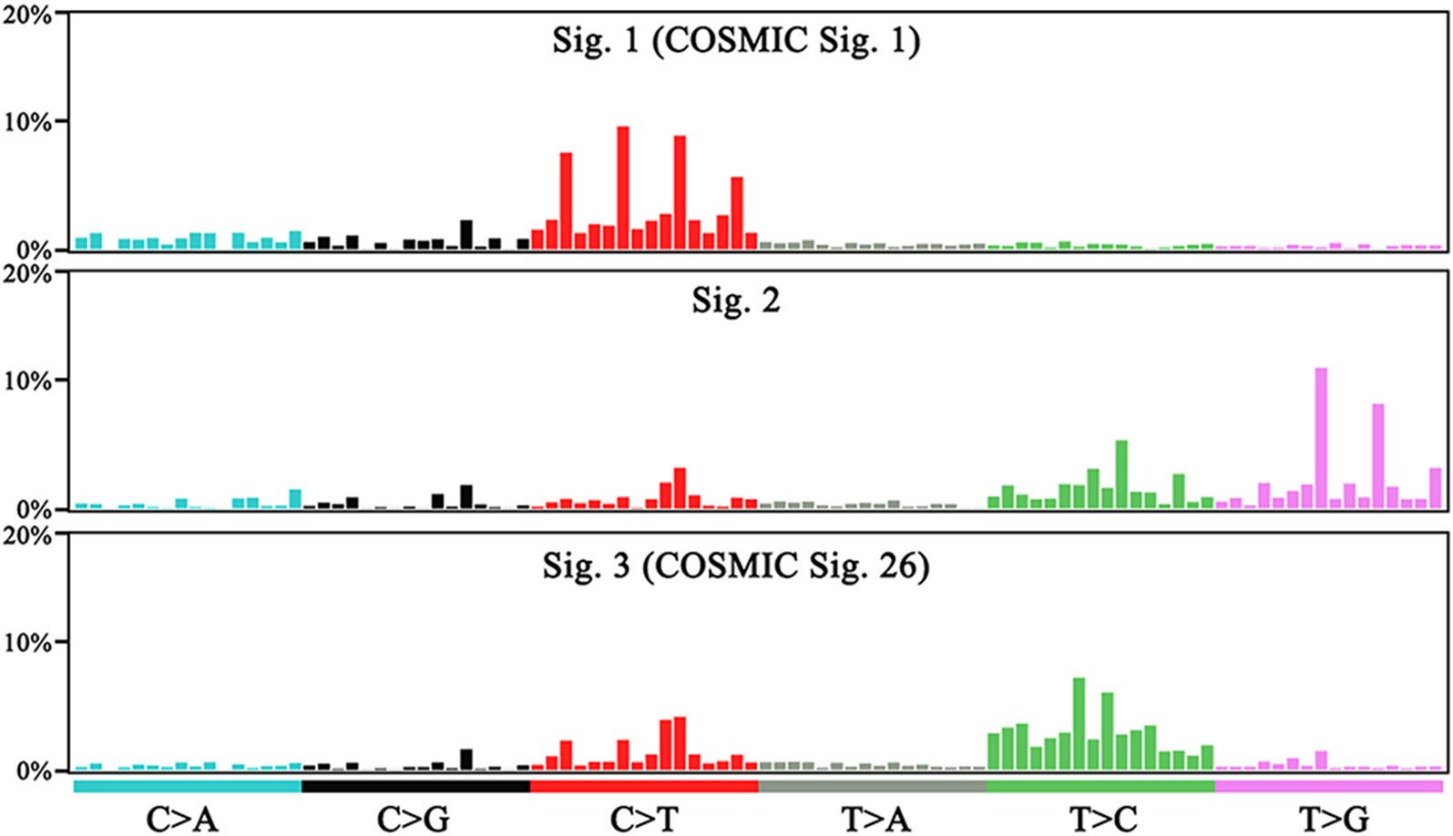
Major mutational signatures were identified according to the alphabetical 96-substitution classifications from 53 pGI-DLBCL patients. The probability bars for the six types of substitutions are displayed in different colors. The mutation types are on the horizontal axes, whereas vertical axes differ between individual signatures for visualization of their patterns and indicate the percentage of mutations attributed to specific mutation types

We performed WES of patient-derived tumor tissue and matched blood DNA. Collectively, 6,588 protein-altering mutational events spanning 3,229 genes were identified from our patient cohort. Of these, 5,489 were missense variants, 171 were in frame insertions or deletions, 394 were frameshift variants, 187 were splice site mutations, 23 were start lost mutations, 13 were stop lost mutations, and 311 were stop gain mutations. The spectrum of the top 40 frequently mutated genes was presented in Fig. 1 and the mutational profile of the entire cohort was summarized in Additional file 2: Table S2. The gene with the highest mutation rate was *IGLL5* (mutated in 47% pGI-DLBCL patients), which is also the top 1 mutated gene reported in HBV-related DLBCL [21]. Other most frequently mutated genes (≥ 15%) included *TP53*, *BTG2*, *P2RY8*, *PCLO*, *HIST1H1E*, *IGHM*, *KMT2D*, *CSMD3*, *MUC16*, *RYR2*, *CCND3*, *DUSP2*, *FAT4*, *IGHJ6*, *CARD11*, *HIST1H1C*, *LRP1B*, *MYC*, *NBPF1*, *SI*. The genome-wide mutational signatures were also characterized according to the 96 possible mutation types [22]. Three highly confident mutational signatures were extracted from our patient cohort. Of these 3 mutation signatures, signatures 1 and 3 were fitted with COSMIC signature 1 and 26, which have been linked to age and defective DNA mismatch repair in cancer, respectively. Meanwhile signature 2, which was mainly characterized by T to G mutations, was not correlated with any COSMIC signature (Fig. 2).

### Potential driver mutations in pGI-DLBCL

In order to identify potential driver mutations in pGI-DLBCL, we compared the mutation profile of our patient cohort with those pathogenic genes associated with human tumors, which have been published and indexed in the COSMIC, MDG125 [23], SMG127 [24], CDG291 datasets [25]. A total of 417 potential driver genes were identified (Table 2). Among these genes, 30 commonly mutated driver genes were found in at least 5 pGI-DLBCL patients, including *TP53*, *P2RY8*, *KMT2D*, *MUC16*, *CSMD3*, *FAT4*, *CCND3*, *HIST1H1C*, *CARD11*, *MYC*, *LRP1B*, *B2M*, *TET2*, *FOXO1*, *EBF1*, *BTG1*, *SETD1B*, *BCR*, *COL3A1*, *DDX3X*, *AHNAK2*, *PIM1*, *ID3*, *DNM2*, *PTPN6*, *FAT1*, *ROBO2*, *NFKBIA*, *BCL7A*, *SGK1*. Next, we used those potential driver genes shared by at least 2 pGI-DLBCL patients to perform gene clustering analysis with the aid of DAVID algorithm. The result revealed that these recurrent driver genes were mainly enriched in pathways related to human cancers, signal transduction, cell metabolism, infection disease and immune regulation. Important signal transduction pathways were substantially affected such as thyroid hormone signaling, central carbon metabolism, HBV infection, FoxO signaling and B cell receptor signaling (Fig. 3 and Additional file 3: Table S3). These results indicated that abnormal signal transduction cascades, altered cell metabolism and virus infection may jointly contribute to the pathogenesis of pGI-DLBCL.

**Table 2:** Potential driver mutations in pGI-DLBCL

#Gene Symbol	Sample	COSMIC	MDG125	SMG127	CDG291	Patient_Number_Count
TP53	P01, P02, P03, P04, P05, P17, P18, P19, P20, P21, P22, P23, P33, P34, P35, P36, P37, P41, P42, P43, P50, P51	oncogene, TSG, fusion	TSG	pancan_fre:42.00%	Yes	22
P2RY8	P09, P12, P13, P17, P18, P19, P20, P25, P27, P29, P30, P38, P52, P53	oncogene, fusion	No	No	No	14
KMT2D	P02, P08, P10, P21, P31, P32, P34, P40, P43, P46, P53	oncogene, TSG	No	No	No	11
MUC16	P01, P03, P09, P10, P13, P24, P36, P45, P50, P51	oncogene	No	No	No	10
CSMD3	P03, P06, P09, P21, P24, P28, P38, P45, P50, P53	TSG	No	No	No	10
FAT4	P01, P06, P08, P09, P19, P20, P27, P50, P52	TSG	No	No	No	9
CCND3	P06, P07, P11, P18, P22, P28, P40, P45, P48	oncogene, fusion	No	No	No	9
HIST1H1C	P06, P14, P18, P26, P27, P34, P38, P53	No	No	pancan_fre:0.60%	Yes	8
CARD11	P01, P20, P40, P43, P45, P46, P48, P52	oncogene	Oncogene	No	No	8
MYC	P04, P14, P22, P26, P33, P34, P37, P50	oncogene, fusion	No	No	No	8
LRP1B	P04, P19, P20, P26, P36, P38, P41, P52	TSG	No	No	No	8
B2M	P06, P09, P11, P20, P27, P31, P38	TSG	TSG	No	Yes	7
TET2	P07, P11, P14, P16, P27, P28, P50	TSG	TSG	pancan_fre:1.60%	Yes	7
FOXO1	P04, P11, P14, P15, P29, P34, P50	oncogene, TSG, fusion	No	No	No	7
EBF1	P01, P04, P17, P18, P26, P32, P53	TSG, fusion	No	No	No	7
BTG1	P06, P25, P27, P38, P39, P40, P42	TSG, fusion	No	No	No	7
SETD1B	P08, P18, P31, P33, P46, P47, P52	TSG	No	No	No	7
BCR	P15, P18, P26, P35, P48, P53	fusion	No	No	No	6
COL3A1	P05, P10, P23, P24, P28, P38	fusion	No	No	No	6
DDX3X	P09, P10, P20, P29, P32, P50	TSG	No	No	Yes	6
AHNAK2	P04, P06, P20, P24, P26, P31	No	No	No	Yes	6
PIM1	P21, P26, P35, P37, P46, P52	oncogene, fusion	No	No	No	6
ID3	P14, P15, P22, P26, P29, P51	TSG	No	No	No	6
DNM2	P01, P13, P20, P28, P38, P40	TSG	No	No	No	6
PTPN6	P06, P11, P12, P25, P38	TSG	No	No	No	5
FAT1	P03, P07, P09, P13, P36	TSG	No	No	No	5
ROBO2	P03, P06, P19, P24, P33	TSG	No	No	No	5
NFKBIA	P12, P18, P43, P50, P53	No	No	No	No	5
BCL7A	P12, P26, P34, P40, P53	fusion	No	No	No	5
SGK1	P04, P06, P18, P25, P28	oncogene	No	No	Yes	5
ZEB2	P06, P13, P31, P48	No	No	No	Yes	4
MEF2B	P08, P34, P47, P52	No	No	No	No	4
PRDM1	P36, P37, P44, P45	TSG	TSG	No	No	4
CD79B	P02, P03, P08, P46	oncogene	No	No	No	4
NFKBIE	P17, P19, P38, P48	TSG	No	No	No	4
SOCS1	P26, P28, P38, P43	TSG	TSG	No	No	4
FAT3	P05, P20, P21, P40		No	No	No	4
CHD4	P07, P24, P35, P40	oncogene	No	No	Yes	4
NCOR2	P02, P20, P36, P42	TSG	No	No	Yes	4
ZFP36L2	P08, P20, P26, P39	No	No	No	Yes	4
DST	P04, P05, P45, P47	No	No	No	Yes	4
KIAA1549	P20, P37, P40, P43	fusion	No	No	No	4
AHNAK	P17, P45, P47, P51	No	No	No	Yes	4
GNAQ	P06, P38, P46, P51	oncogene	Oncogene	No	No	4
TBL1XR1	P06, P18, P26, P51	oncogene, TSG, fusion	No	pancan_fre:0.80%	Yes	4
HLA-B	P13, P19, P24, P27	No	No	No	Yes	4
BRAF	P01, P04, P06, P53	oncogene, fusion	Oncogene	pancan_fre:1.50%	Yes	4
ACTB	P06, P17, P20, P35	No	No	No	Yes	4
PLEC	P06, P11, P28, P40	No	No	No	Yes	4
SYNE1	P04, P06, P33, P34	No	No	No	Yes	4
DCC	P03, P24, P36, P52		No	No	No	4
ROS1	P01, P20, P24, P45	oncogene, fusion	No	No	No	4
ARID1A	P04, P11, P18, P22	TSG, fusion	TSG	pancan_fre:5.40%	Yes	4
TNFRSF14	P06, P11, P14, P25	TSG	No	No	No	4
STAT3	P04, P18, P19, P48	oncogene	No	No	Yes	4
PIK3CD	P13, P16, P20	No	No	No	No	3
FAM135B	P06, P20, P38		No	No	No	3
TRIO	P04, P36, P40	No	No	No	Yes	3
TRIM24	P03, P20, P50	oncogene, TSG, fusion	No	No	No	3
UBR5	P04, P20, P43	TSG	No	No	No	3
FAM47C	P04, P17, P34		No	No	No	3
LRRK2	P09, P42, P52	No	No	pancan_fre:2.80%	Yes	3
GRIN2A	P01, P04, P20	TSG	No	No	No	3
FBN2	P01, P09, P20	No	No	No	Yes	3
NEB	P01, P36, P51	No	No	No	Yes	3
IRS2	P02, P50, P53	No	No	No	Yes	3
PRKCD	P06, P11, P24	No	No	No	Yes	3
ACTG1	P06, P14, P26	No	No	No	Yes	3
KALRN	P20, P31, P43	No	No	No	Yes	3
BIRC6	P06, P09, P20	oncogene, fusion	No	No	No	3
CLTC	P16, P20, P50	TSG, fusion	No	No	Yes	3
APC	P06, P18, P36	TSG	TSG	pancan_fre:7.30%	Yes	3
PTEN	P01, P09, P35	TSG	TSG	pancan_fre:9.70%	Yes	3
CXCR4	P01, P26, P50	oncogene	No	No	No	3
JMJD1C	P03, P08, P12	No	No	No	Yes	3
FAS	P06, P09, P18	TSG	No	No	No	3
BCL6	P05, P43, P52	oncogene, fusion	No	No	No	3
PCBP1	P09, P44, P46		No	pancan_fre:0.30%	Yes	3
BCL11B	P07, P11, P12	oncogene, TSG, fusion	No	No	No	3
PTPRB	P01, P36, P50	TSG	No	No	No	3
CIITA	P11, P25, P40	TSG, fusion	No	No	No	3
HGF	P09, P36, P48	No	No	pancan_fre:1.70%	Yes	3
IRF4	P08, P38, P42	oncogene, TSG, fusion	No	No	No	3
NIN	P17, P27, P36	fusion	No	No	Yes	3
RARA	P10, P33, P48	oncogene, fusion	No	No	No	3
TRRAP	P20, P36, P50	oncogene	No	No	No	3
MAP2K1	P12, P28, P50	oncogene	Oncogene	No	No	3
KMT2C	P05, P11, P15	TSG	No	No	No	3
PABPC1	P25, P26, P32	oncogene, TSG	No	No	Yes	3
PIK3CB	P32, P53	oncogene	No	No	Yes	2
CBLB	P26, P52	TSG	No	No	No	2
MDN1	P09, P53	No	No	No	Yes	2
RAB11FIP5	P07, P20	No	No	No	Yes	2
FIP1L1	P01, P15	fusion	No	No	No	2
CFH	P09, P20	No	No	No	Yes	2
KDM6B	P26, P53	No	No	No	Yes	2
MYCN	P25, P27	oncogene	No	No	No	2
CAMTA1	P37, P51	TSG, fusion	No	No	No	2
TCF7	P41, P44	No	No	No	Yes	2
PDGFRA	P20, P40	oncogene, fusion	Oncogene	pancan_fre:1.90%	Yes	2
TET1	P09, P20	oncogene, TSG, fusion	No	No	No	2
ARHGAP32	P01, P04	No	No	No	Yes	2
SFRP4	P09, P12	TSG	No	No	No	2
PRRC2A	P20, P50	No	No	No	Yes	2
NTRK2	P04, P25	No	No	No	No	2
HSP90AB1	P11, P20	fusion	No	No	Yes	2
KRAS	P25, P28	oncogene	Oncogene	pancan_fre:6.70%	Yes	2
PCM1	P06, P24	fusion	No	No	Yes	2
SMARCA4	P15, P28	TSG	TSG	No	Yes	2
CHD8	P38, P50	No	No	No	Yes	2
NCOR1	P03, P32	TSG	TSG	pancan_fre:2.20%	Yes	2
ZFP36L1	P26, P46	No	No	No	Yes	2
MKI67	P17, P45	No	No	No	Yes	2
RGPD3	P45, P48		No	No	No	2
FBXO11	P07, P51	TSG	No	No	Yes	2
LRIG3	P01, P20	TSG, fusion	No	No	No	2
NFATC2	P08, P43	oncogene, fusion	No	No	No	2
KIT	P10, P23	oncogene	Oncogene	pancan_fre:1.40%	Yes	2
CREBBP	P09, P20	oncogene, TSG, fusion	TSG	No	No	2
TCL1A	P07, P25	oncogene, fusion	No	No	No	2
MSH3	P12, P42	No	No	No	No	2
SF3B1	P01, P11	oncogene	Oncogene	pancan_fre:1.30%	Yes	2
PRKCB	P04, P13		No	No	No	2
ZNF91	P24, P40	No	No	No	Yes	2
BCLAF1	P09, P53		No	No	Yes	2
MAP3K4	P11, P13	No	No	No	Yes	2
FGFR4	P45, P50	oncogene	No	No	No	2
FGFR2	P45, P52	oncogene, fusion	Oncogene	pancan_fre:1.50%	Yes	2
PRPF8	P01, P09	No	No	No	Yes	2
SPEN	P11, P38	TSG	No	No	Yes	2
SPEG	P45, P53	No	No	No	Yes	2
PDE4DIP	P03, P38	fusion	No	No	No	2
AFF3	P01, P17	oncogene, fusion	No	No	No	2
SALL4	P40, P50	oncogene	No	No	No	2
ANKRD11	P04, P35	No	No	No	Yes	2
TFDP1	P26, P42	No	No	No	Yes	2
INPP4B	P36, P50	No	No	No	No	2
MICAL1	P09, P40	No	No	No	Yes	2
SIN3A	P15, P34	No	No	pancan_fre:1.10%	Yes	2
HLA-A	P12, P18	fusion	No	No	Yes	2
TFEB	P04, P28	oncogene, fusion	No	No	No	2
KIAA1109	P20, P40	No	No	No	Yes	2
TNFAIP3	P11, P36	TSG	TSG	No	No	2
TP63	P09, P11	oncogene, TSG	No	No	No	2
PTPRD	P40, P45	TSG	No	No	No	2
CLTCL1	P20, P48	TSG, fusion	No	No	Yes	2
ZMYM3	P09, P20	TSG	No	No	No	2
MGA	P01, P41	No	No	No	Yes	2
NSD1	P48, P51	fusion	No	pancan_fre:2.40%	Yes	2
CSF1R	P20, P42	oncogene	Oncogene	No	No	2
MEGF6	P11, P45	No	No	No	Yes	2
HIST1H3B	P01, P26	oncogene	Oncogene	No	No	2
ADCY1	P03, P20	No	No	No	Yes	2
RET	P17, P27	oncogene, fusion	Oncogene	No	No	2
EPHA7	P26, P36		No	No	No	2
EPHA3	P20, P51		No	pancan_fre:2.10%	Yes	2
RBM15	P04, P09	fusion	No	No	No	2
ZNF521	P08, P09	oncogene, fusion	No	No	No	2
CNTNAP2	P09, P35	TSG	No	No	No	2
RASA1	P28, P51	No	No	No	Yes	2
PTPRC	P26, P31	TSG	No	No	No	2
CAD	P20, P37	No	No	No	Yes	2
EPS15	P32, P50	TSG, fusion	No	No	No	2
EXT2	P05, P20	TSG	No	No	No	2
RAG1	P24, P38	No	No	No	Yes	2
CDH10	P03, P12	TSG	No	No	No	2
ZFHX3	P01, P20	TSG	No	No	Yes	2
MTOR	P07, P51	oncogene	No	pancan_fre:3.00%	Yes	2
EP300	P06, P09	TSG, fusion	TSG	pancan_fre:2.50%	Yes	2
CNBD1	P06, P12		No	No	No	2
ABCB1	P24, P42	No	No	No	Yes	2
CTNNA2	P09, P25	oncogene	No	No	No	2
NOTCH1	P33, P37	oncogene, TSG, fusion	TSG	pancan_fre:3.10%	Yes	2
IKBKB	P09, P27	oncogene	No	No	No	2
MYO5A	P01, P38	fusion	No	No	No	2
STRN	P20, P50	fusion	No	No	No	2
NRG1	P20, P53	TSG, fusion	No	No	No	2
MALT1	P28, P48	oncogene, fusion	No	No	No	2
PHF6	P08, P20	TSG	TSG	pancan_fre:0.80%	Yes	2
NAV3	P04, P45	No	No	pancan_fre:4.60%	Yes	2
MYCBP2	P04, P43	No	No	No	Yes	2
NBEA	P48, P53		No	No	Yes	2
HSP90AA1	P04, P26	fusion	No	No	No	2
CHD7	P31, P37	No	No	No	Yes	2
PIK3CG	P52	No	No	pancan_fre:1.70%	Yes	1
HIST1H4I	P14	fusion	No	No	No	1
HSPA8	P04	No	No	No	Yes	1
NUP98	P20	oncogene, fusion	No	No	Yes	1
XPA	P46	TSG	No	No	No	1
CEP89	P04	fusion	No	No	No	1
XPO1	P28	oncogene	No	No	No	1
CSDE1	P51	No	No	No	Yes	1
TTK	P09	No	No	No	Yes	1
COL1A1	P26	fusion	No	No	No	1
ZEB1	P52	oncogene	No	No	No	1
ITGAV	P13		No	No	No	1
ZNF703	P14	No	No	No	Yes	1
ERBB2IP	P14	No	No	No	Yes	1
ARHGEF12	P20	TSG, fusion	No	No	No	1
MUC1	P29	fusion	No	No	No	1
EWSR1	P20	oncogene, fusion	No	No	Yes	1
AHCTF1	P26	No	No	No	Yes	1
RPL22	P09	TSG, fusion	No	pancan_fre:1.00%	Yes	1
SIX2	P22	oncogene	No	No	No	1
PRX	P20	No	No	pancan_fre:0.90%	Yes	1
ARID2	P06	TSG	TSG	No	Yes	1
SET	P20	oncogene, fusion	No	No	No	1
ELK4	P36	oncogene, fusion	No	No	No	1
TRIM7	P46	No	No	No	Yes	1
FBXW7	P05	TSG	TSG	pancan_fre:3.00%	Yes	1
TGFBR2	P11	TSG	No	pancan_fre:1.10%	Yes	1
SH3PXD2A	P20	No	No	No	Yes	1
SVIL	P20	No	No	No	Yes	1
PHLDA1	P21	No	No	No	Yes	1
NBPF10	P28	No	No	No	Yes	1
PBX1	P50	oncogene, fusion	No	No	No	1
ARHGAP35	P20	No	No	pancan_fre:2.50%	Yes	1
PTCH1	P33	TSG	TSG	No	No	1
CUL1	P23	No	No	No	Yes	1
CDX2	P20	TSG, fusion	No	No	No	1
PTPN13	P12	TSG	No	No	Yes	1
IRS4	P09	oncogene, TSG	No	No	No	1
DMD	P06	No	No	No	Yes	1
PPM1D	P09	oncogene	No	No	No	1
SRSF2	P14	oncogene	Oncogene	No	No	1
RALGAPA1	P17	No	No	No	Yes	1
EIF1AX	P04		No	No	No	1
MED12	P11	TSG	Oncogene	No	Yes	1
NTRK3	P45	oncogene, fusion	No	No	No	1
MED13	P20	No	No	No	Yes	1
ARHGAP26	P21	TSG, fusion	No	No	No	1
SRGAP3	P01	fusion	No	No	No	1
ACSL6	P01	fusion	No	No	No	1
FLI1	P01	oncogene, fusion	No	No	No	1
CHD2	P28	TSG	No	No	No	1
POLG	P20	TSG	No	No	No	1
DDX5	P23	oncogene, fusion	No	No	Yes	1
MN1	P52	oncogene, fusion	No	No	Yes	1
PRDM16	P24	oncogene, fusion	No	No	No	1
POT1	P53	TSG	No	No	No	1
ARHGAP5	P20	oncogene	No	No	No	1
SOS1	P51	No	No	No	Yes	1
KIF20B	P20	No	No	No	Yes	1
TSHZ2	P47	No	No	pancan_fre:1.80%	No	1
EIF3E	P45	TSG, fusion	No	No	No	1
BCL2L12	P39	oncogene	No	No	No	1
KAT6A	P41	oncogene, fusion	No	No	No	1
CDH11	P27	TSG, fusion	No	No	No	1
BAP1	P53	TSG	TSG	pancan_fre:2.00%	Yes	1
UBE4A	P20	No	No	No	Yes	1
JAK2	P09	oncogene, fusion	Oncogene	No	Yes	1
N4BP2	P26	TSG	No	No	No	1
GRM3	P13	oncogene	No	No	No	1
ZNF384	P06	fusion	No	No	No	1
AKAP9	P01	fusion	No	No	Yes	1
EEF1A1	P08	No	No	No	Yes	1
PBRM1	P20	TSG	TSG	pancan_fre:5.40%	Yes	1
ERC1	P48	fusion	No	No	No	1
ERG	P36	oncogene, fusion	No	No	No	1
MYOD1	P36	oncogene	No	No	No	1
CDK12	P25	TSG	No	pancan_fre:1.50%	Yes	1
A1CF	P45	oncogene	No	No	No	1
WT1	P23	oncogene, TSG, fusion	TSG	pancan_fre:1.00%	Yes	1
BARD1	P31	TSG	No	No	Yes	1
BAZ1A	P31	TSG	No	No	No	1
FN1	P01	No	No	No	Yes	1
FUBP1	P51	oncogene	TSG	No	No	1
PRRX1	P51	fusion	No	No	No	1
ATR	P25	TSG	No	pancan_fre:2.40%	Yes	1
BRIP1	P53	TSG	No	No	No	1
FLT1	P01	No	No	No	No	1
FANCF	P40	TSG	No	No	No	1
PTK6	P12	oncogene, TSG	No	No	No	1
MSH6	P20	TSG	TSG	No	No	1
SPECC1	P45	fusion	No	No	No	1
PRKCI	P01	No	No	No	No	1
MATK	P48	No	No	No	Yes	1
ACKR3	P50	oncogene, fusion	No	No	No	1
ERBB3	P32	oncogene	No	No	No	1
IDH2	P42	oncogene	Oncogene	pancan_fre:0.80%	Yes	1
FGFR3	P13	oncogene, fusion	Oncogene	pancan_fre:1.00%	Yes	1
FGFR1	P51	oncogene, fusion	No	No	No	1
AFF4	P31	oncogene, fusion	No	No	No	1
MAP1 B	P08	No	No	No	Yes	1
EPB41L3	P04	No	No	No	Yes	1
TPR	P43	fusion	No	No	Yes	1
GNAS	P19	oncogene	Oncogene	No	Yes	1
RBMX	P53	No	No	No	Yes	1
AFF1	P06	fusion	No	No	No	1
CDKN2C	P26	TSG	No	pancan_fre:0.20%	Yes	1
WHSC1L1	P04	oncogene, fusion	No	No	Yes	1
GOT2	P47	No	No	No	Yes	1
LYN	P11	No	No	No	Yes	1
MGMT	P06	TSG	No	No	No	1
PMS1	P20		No	No	No	1
PMS2	P20	TSG	No	No	No	1
LHFP	P14	fusion	No	No	No	1
AMER1	P52	TSG	No	No	No	1
NACA	P09	fusion	No	No	No	1
FGF4	P13	No	No	No	No	1
FGF3	P35	No	No	No	No	1
HOXD11	P40	oncogene, fusion	No	No	No	1
SMCHD1	P03	No	No	No	Yes	1
JAZF1	P19	fusion	No	No	No	1
BCOR	P40	TSG, fusion	TSG	No	Yes	1
ADAM10	P03	No	No	No	Yes	1
G3BP1	P09	No	No	No	Yes	1
BCL10	P05	TSG, fusion	No	No	No	1
CDKN1B	P40	TSG	No	pancan_fre:0.70%	Yes	1
SETBP1	P12	oncogene, fusion	Oncogene	pancan_fre:2.20%	No	1
AKT1	P14	oncogene	Oncogene	pancan_fre:0.90%	Yes	1
PSIP1	P50	oncogene, fusion	No	No	No	1
CCDC6	P36	TSG, fusion	No	No	No	1
ARHGEF10	P25	TSG	No	No	No	1
REL	P19	oncogene	No	No	No	1
COL2A1	P17	fusion	No	No	No	1
TSC1	P12	TSG	TSG	No	No	1
SMC3	P26	No	No	pancan_fre:1.20%	Yes	1
ARID5B	P37	No	No	pancan_fre:1.60%	Yes	1
IGF1R	P15	No	No	No	No	1
HNF1A	P20	TSG	TSG	No	No	1
E2F3	P26	No	No	No	No	1
ARHGEF6	P51	No	No	No	Yes	1
CDH1	P48	TSG	TSG	pancan_fre:2.50%	Yes	1
KIFC3	P01	No	No	No	Yes	1
ARHGEF10L	P21	TSG	No	No	No	1
NEK8	P17	No	No	No	Yes	1
FAM129B	P20	No	No	No	Yes	1
IL7R	P36	oncogene	No	No	No	1
MYH9	P10	TSG, fusion	No	No	Yes	1
CYLD	P20	TSG	TSG	No	Yes	1
CASC5	P09	TSG, fusion	No	No	No	1
NUTM1	P48	oncogene, fusion	No	No	No	1
SOX17	P11	No	No	pancan_fre:0.30%	Yes	1
BRCA1	P11	TSG	TSG	pancan_fre:1.90%	Yes	1
BRCA2	P20	TSG	TSG	pancan_fre:2.70%	Yes	1
WNK2	P53	TSG	No	No	No	1
P4HB	P26	No	No	No	Yes	1
ARNT	P53	oncogene, TSG, fusion	No	No	No	1
BCL3	P07	oncogene, fusion	No	No	No	1
RNF213	P20	fusion	No	No	Yes	1
DOCK2	P32	No	No	No	Yes	1
09-Sep	P31	fusion	No	No	No	1
05-Sep	P12	fusion	No	No	No	1
DCAF12L2	P23		No	No	No	1
NEDD4L	P20	No	No	No	Yes	1
RAP1GDS1	P38	oncogene, fusion	No	No	No	1
RPP38	P20	No	No	No	Yes	1
CTNND2	P43	oncogene	No	No	No	1
ATRX	P19	TSG	TSG	pancan_fre:2.80%	Yes	1
RAD51B	P44	TSG, fusion	No	No	No	1
TP53BP1	P20	No	No	No	Yes	1
PICALM	P20	fusion	No	No	No	1
BCL2	P26	oncogene, fusion	Oncogene	No	No	1
ASXL2	P40	TSG	No	No	No	1
SMC1A	P35	TSG	No	pancan_fre:1.50%	Yes	1
TLR4	P43	No	No	pancan_fre:1.90%	Yes	1
KDM6A	P50	oncogene, TSG	TSG	pancan_fre:2.00%	Yes	1
MET	P06	oncogene	Oncogene	No	No	1
DNM3	P36	No	No	No	Yes	1
BCL11A	P20	oncogene, fusion	No	No	No	1
GATA3	P20	oncogene, TSG	TSG	pancan_fre:3.20%	Yes	1
RPN1	P45	fusion	No	No	No	1
EPPK1	P11	No	No	pancan_fre:1.40%	Yes	1
AXL	P20	No	No	No	No	1
CBL	P26	oncogene, TSG, fusion	Oncogene	No	No	1
PRDM2	P46	TSG	No	No	Yes	1
GIGYF2	P03	No	No	No	Yes	1
NR4A2	P12	No	No	No	Yes	1
MITF	P38	oncogene	No	No	No	1
RPTOR	P08	No	No	No	No	1
CNOT3	P46	TSG	No	No	Yes	1
BRD3	P20	oncogene, fusion	No	No	No	1
SPTAN1	P43	No	No	No	Yes	1
PPFIBP1	P20	fusion	No	No	No	1
MKL1	P50	oncogene, TSG, fusion	No	No	No	1
FANCD2	P50	TSG	No	No	No	1
ZBTB16	P06	TSG, fusion	No	No	No	1
DOCK4	P47	No	No	No	Yes	1
SND1	P50	oncogene, fusion	No	No	No	1
ERCC3	P45	TSG	No	No	No	1
USP6	P07	oncogene, fusion	No	No	No	1
HIP1	P52	oncogene, fusion	No	No	No	1
INTS1	P32	No	No	No	Yes	1
TGOLN2	P38	No	No	No	Yes	1
IDH1	P14	oncogene	Oncogene	pancan_fre:1.50%	Yes	1
PTPRK	P39	TSG, fusion	No	No	No	1
GMPS	P40	fusion	No	No	No	1
ATIC	P03	fusion	No	No	No	1
FOXA2	P20	No	No	pancan_fre:0.50%	Yes	1
CDKN2A	P22	TSG	TSG	pancan_fre:3.60%	Yes	1
SKI	P45	oncogene	No	No	No	1
CCR7	P11	oncogene	No	No	No	1
FOSL2	P06	No	No	No	Yes	1
PWWP2A	P51	fusion	No	No	No	1
DDR2	P09	oncogene	No	No	No	1
CD274	P07	TSG, fusion	No	No	No	1
CDH17	P32	oncogene	No	No	No	1
FANCA	P26	TSG	No	No	Yes	1
ARID1B	P38	TSG	TSG	No	No	1
NIPBL	P09	No	No	No	Yes	1
KMT2A	P19	oncogene, fusion	No	No	No	1
ANKRD6	P01	No	No	No	Yes	1
CTNND1	P03		No	No	Yes	1
MACF1	P11	No	No	No	Yes	1
PABPC4	P27	No	No	No	Yes	1
PREX2	P26	oncogene	No	No	No	1
ZNRF3	P04	TSG	No	No	No	1
ETV1	P20	oncogene, fusion	No	No	No	1
ETV5	P09	oncogene, fusion	No	No	No	1
TAF1	P06	No	No	pancan_fre:2.30%	Yes	1
HOXA11	P14	oncogene, TSG, fusion	No	No	No	1
ABL2	P01	oncogene, fusion	No	No	No	1
POLD1	P20	TSG	No	No	No	1
HMGA2	P13	oncogene, fusion	No	No	No	1
MSN	P04	fusion	No	No	Yes	1
ZRSR2	P22	TSG	No	No	No	1

**Fig. 3:**
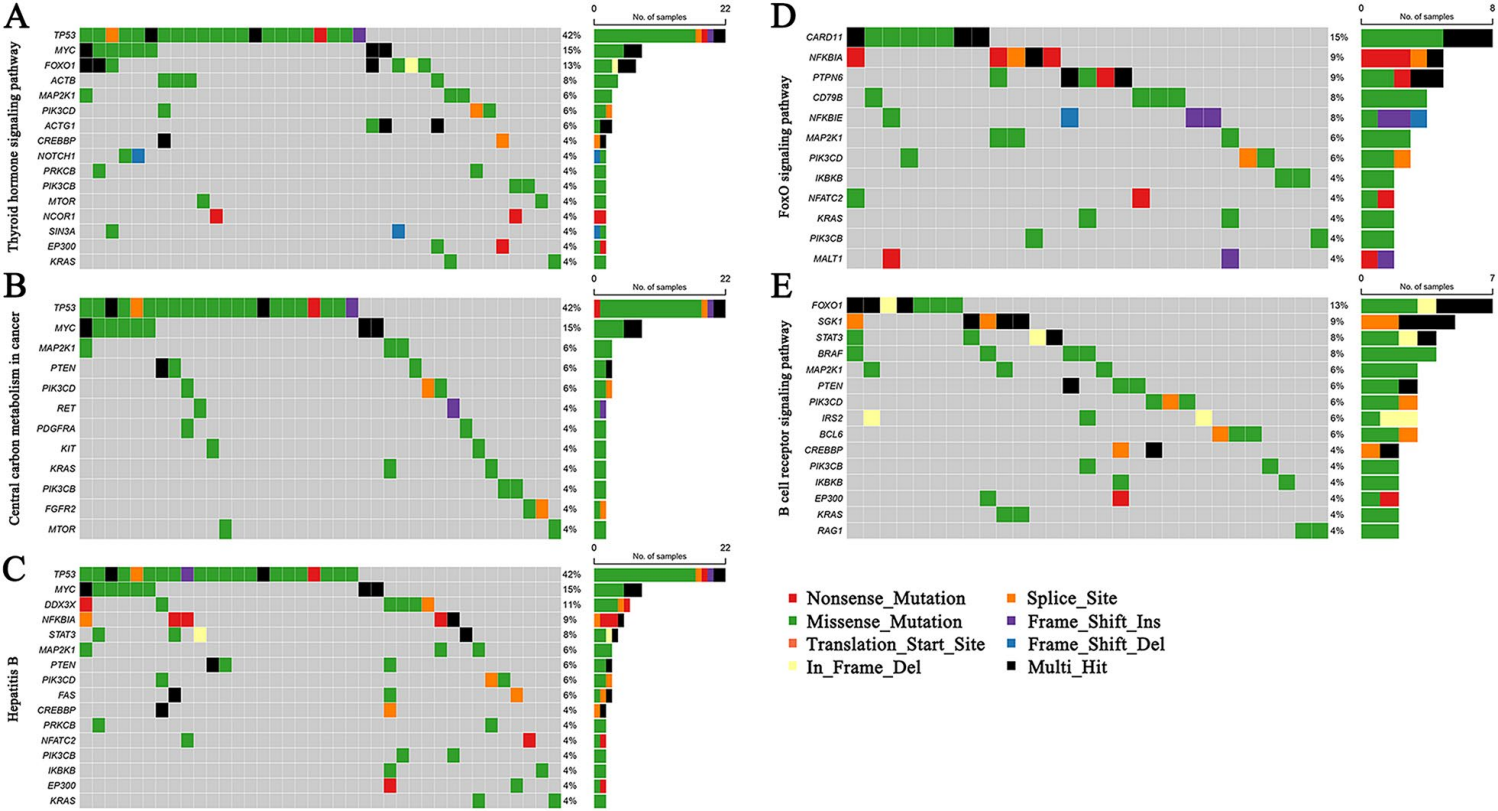
Heatmap of potential oncogenic pathways affected by exonic mutations in 53 pGI-DLBCL patients. **A** Thyroid hormone signaling pathway. **B** Central carbon metabolism in cancer. **C** Hepatitis **B**. **D** FoxO signaling pathway. **E** B cell receptor signaling pathway. The mutation rate of each gene is displayed on the right of each row. The histogram on the right shows the number of mutations in each gene

### Associations between clinicopathological characteristics and exonic mutations in pGI-DLCBL pateints

**Fig. 4:**
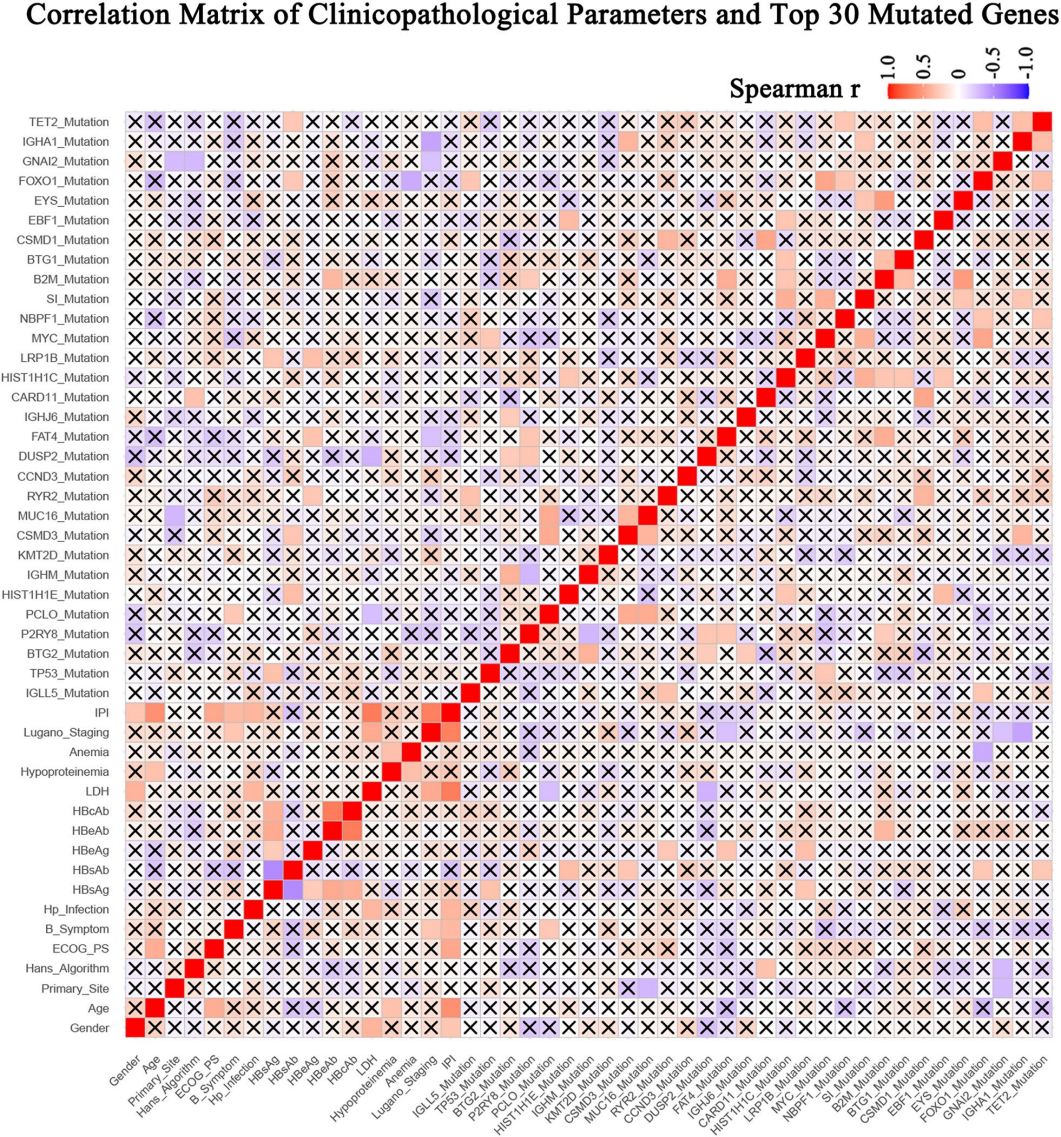
The Spearman correlation matrix between major clinicopathological parameters and the status of top 30 mutated genes across 53 pGI-DLBCL patients. The correlations were obtained by deriving Spearman's correlation coefficients. Red represents a positive correlation and blue represents a negative correlation. The cross mark in each box denotes that the correlation did not reach statistical significance

**Fig. 5:**
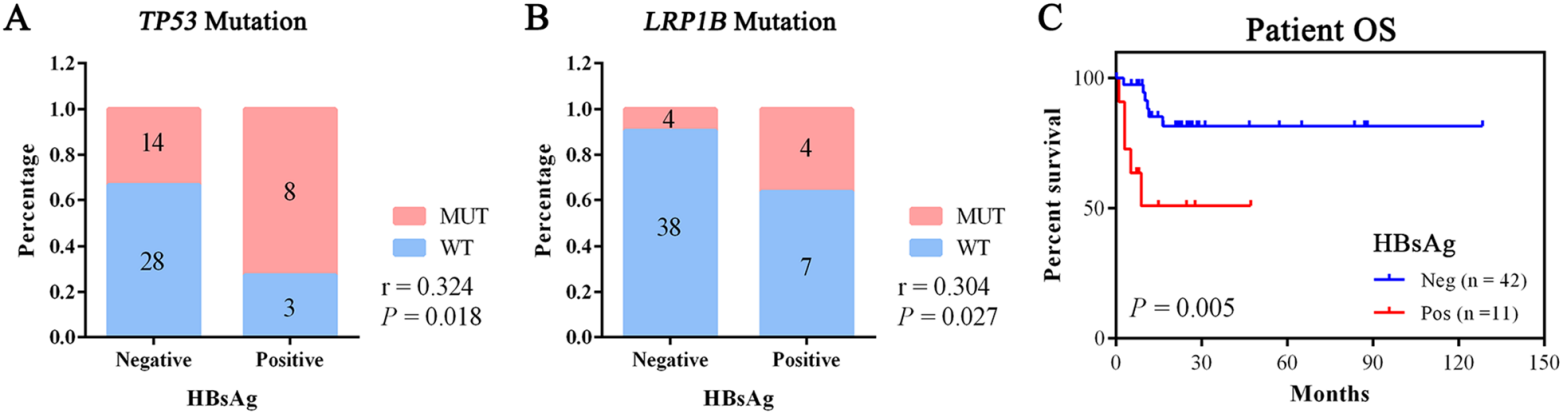
HBV infection was associated with certain mutations and patient OS in pGI-DLBCL. **A**, **B** The bar graph indicates the Spearman’s correlation between HBsAg and *TP53* (**A**) or *LRP1B* (**B**) mutation. The stacked percentage for each group is shown and the number in the bar denotes patient number count for each group. **C** OS for pGI-DLBCL patients stratified by HBsAg status

We analyzed the correlations between the status of top 30 mutated genes and the clinicopathological characteristics, such as age, gender, Hp or HBV infection, LDH level, Eastern Cooperative Oncology Group (ECOG) score, B symptoms, International Prognostic Index (IPI), tumor stage, etc. The result was displayed in Fig. 4, and the correlations with statistical significance were summarized in Additional file 4: Table S4. Interestingly, younger patients tended to have *FAT4* and *FOXO1* mutations, and patients with non-GCB tumors were correlated with *CARD11* mutations. Hp infection showed no association with any parameter, however, HBV infection seemed to be related to certain mutations in pGI-DLBCL, as positive HBsAg was significantly associated with the mutations of *TP53* and *LRP1B*, two important tumor suppressor genes (TSGs) reported in many human cancers (Fig. 5A, B). Moreover, HBsAg positive pGI-DLBCL patients have a significant shorter overall survival (OS), when compared to those without HBV infection (Fig. 5C). These results indicated that genetic mutations in pGI-DLBCL patients were associated with certain clinicopathological parameters, and HBV infection could possibly cause worse prognosis due to mutation in TSGs.

### Mutations correlated with patient survival in pGI-DLBCL

**Fig. 6:**
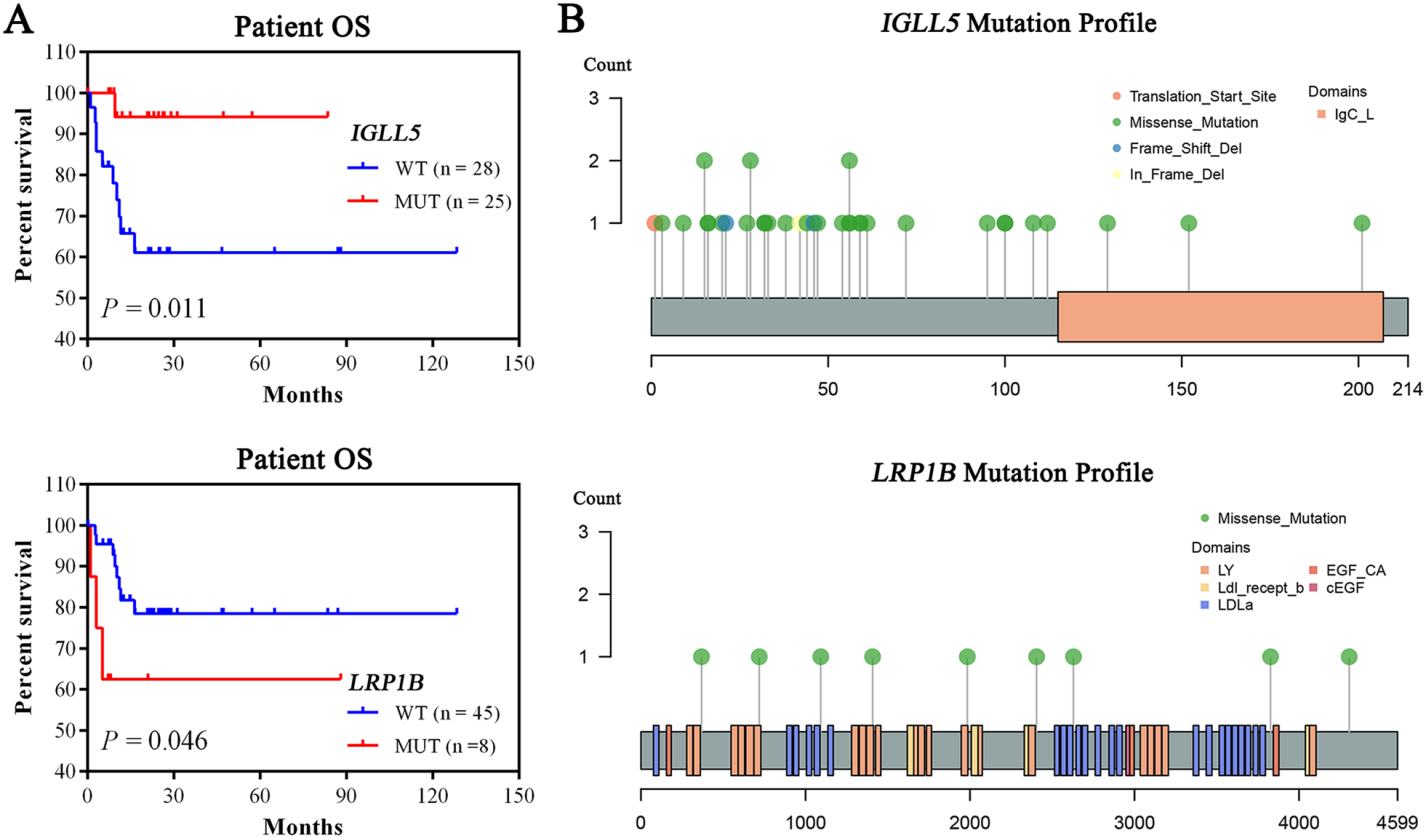
*IGLL5* and *LRP1B* mutations were correlated with OS in pGI-DLBCL. **A** OS for pGI-DLBCL patients stratified by *IGLL5* (upper panel) or *LRP1B* (lower panel) mutation. **B** Lollipop plots with the distribution of somatic mutations on the linear protein and domains of *IGLL5* (upper panel) or *LRP1B* (lower panel) in pGI-DLBCL. Each lollipop denotes a unique mutation location, and its height represents the number of observed mutations. Colored bars indicate the individual protein domains. The type of the mutation is indicated in the legend

In order to find potential genetic mutations with predictive value for patient OS, we performed survival analysis with the top 30 mutated genes in our pGI-DLBCL patient cohort. Most of the mutated genes were not significantly associated with patient OS. However, we did observe that patients with *IGLL5* mutations presented with a better OS, and *LRP1B* mutations led to a shorter OS (Fig. 6A). A large proportion of the mutations in *IGLL5* were missense variants located at its N-terminus uncharacterized domains, and the *LRP1B* mutations were all missense variants evenly distributed across the entire protein structure **(**Fig. 6B and Additional file 5: Table S5). How these mutations affect individual gene function and the patient survival needs further exploration.

## Discussion

In the current study, we performed WES of the largest cohort of pGI-DLBCL to date and identified putative cancer driver mutations and their enriched signaling pathways. We also revealed that HBV infection had an impact on the exonic mutation profile pGI-DLBCL, and mutations of *IGLL5* and *LRP1B* genes could predict patient survival, which to our knowledge, was previously unreported by others.

In accordance with the previous reports [17], our analysis of the pGI-DLBCL exome confirmed the high prevalence of mutations in the cell cycle and apoptosis regulatory pathway, with potential tumor driver mutations in *TP53* (22/53), *CCND3* (9/53) and *MYC* (8/53) in over 60% patients. *TP53* mutations displayed a significantly increased frequency and *MYD88* (0/53), *NFKBIE* (4/53) or *CD79B* (4/53) mutations were less or not found in our pGI-DLBCL cohort, suggesting that the pathogenesis of pGI-DLBCL were different from the nodal or other extranodal DLBCL, which relies on an activated NF-κB signaling pathway due to the common mutations in the above mentioned *MYD88*, *NFKBIE*, or *CD79B* genes [26]. Furthermore, mutation frequencies of *MUC16* (10/53), *CSMD3* (10/53), *RYR2* (10/53), *FAT4* (9/53), *TET2* (7/53), *EBF1* (7/53) and *SETD1B* (7/53), which functions at the transcriptional regulation, epigenetic modification or either cellular attachment, were also increased compared to those in common DLBCL according to COSMIC database. Third, we also identified a relatively large proportion of gene mutations, like *P2RY8* (14/53), *LRP1B* (8/53), *B2M* (7/53), *BCR* (6/53), that seldom mentioned by other DLBCL sequencing studies but may probably become the oncogenic events by modulating the B cell migratory behavior and signaling activation [27, 28]. Therefore, we hypothesized that the mutation signature of pGI-DLBCL was different from other DLBCL subtypes, and the potential oncogenic driver mutations should be validated by further research.

Another important finding of our study was that HBV infection may affect the mutation spectrum of pGI-DLBCL. We showed that the oncogenic driver mutations were significantly enriched in the HBV regulatory pathway, and patients with positive HBsAg status had a relatively shorter OS and were more likely to carry *TP53* and *LRP1B* mutations, both of which are supposed to function as TSGs during lymphomagenesis process. Previous studies have shown that HBV infection could cause an enhanced rate of mutagenesis and a distinct set of mutation targets in common DLBCL genome [21]. It is worth mentioning that the three genes, namely *IGLL5*, *TP53* and *BTG2*, are among the top 5 most mutated genes among their and our WES data. Interestingly, *LRP1B* have been described as a common target gene for HBV integration in liver cancer [29]. In addition, meta-analysis also revealed that patients infected with HBV had a higher risk of developing DLBCL, and those HBsAg-positive DLBCL patients tended to be diagnosed at a younger age with a more advanced clinical stage and worse outcome [30, 31]. Our study presents the first genomic analysis reinforcing the relationship between HBV infection and the mutation signature of pGI-DLBCL. However, further investigations are needed to verify the interactive mechanism between HBV integration and pGI-DLBCL genome, and how the HBV-related mutations affect the pathogenesis and development processes of pGI-DLBCL disease.

Highlighting the clinical significance of our finding, we identified that two recurrent mutations, *IGLL5* and *LRP1B*, could serve as prognostic biomarkers for pGI-DLBCL patients. Although the function of *IGLL5* has not been clarified, pervious reports have shown that it was commonly mutated in DLBCL [32, 33] and is homologous to *IGLL1*, a gene which is critical for B-cell development [34]. In chronic lymphocytic leukaemia (CLL), *IGLL5* mutations were associated with a trend towards decreased overall gene expression, and patients bearing *IGLL5* mutations were suggestive for the low-risk of CLL [35], which to some extent, was consistent to our result showing that *IGLL5* mutated pGI-DLBCL patients had a better OS. On the other hand, *LRP1B* is giant membrane molecule that is among the most altered genes in human malignancies [36]. Functional studies have confirmed that LRP1B expression in cancer cells could reduce in vitro cell proliferation and migration abilities, and also suppress in vivo tumorigenicity in mouse models [37–40]. Genetic alteration events, such as deletions, point mutations or frameshift mutations commonly led to the inactivation of this TSG [41–43]. Therefore, it is speculated that *LRP1B* mutations found in our pGI-DLBCL cohort was associated with the impairment of its gene function, which could cause inferior result on disease progression. Despite we first propose that mutations of *IGLL5* and *LRP1B* were significantly related to the survival of pGI-DLBCL patients, there is still a lack of detailed information on how the mutations affect their expression and/or functional role. Some research suggested that Tumor mutation burden estimated by cancer gene panels (CGPs) could be a potential predictor for prognostic stratification of Chinese DLBCL patients [44]. However, *IGLL5* and *LRP1B* discovered in our study as potential biomarkers for the therapeutics or prognosis of pGI-DLBCL remain to be fully elucidated.

In summary, we performed a comprehensive analysis of the exonic mutation profile of the largest pGI-DLBCL cohort to date, which was characterized by an increased mutation frequency in *TP53* and *MYC*, and a decrease rate or absence of *MYD88* or *CD79B* alteration. We also revealed that HBV infection was related to the mutational signature and patient prognosis of pGI-DLBCL. *IGLL5* and *LRP1B* could serve as predictive biomarkers for patient survival. Our study provides a deeper understanding of the genomic information of pGI-DLBCL and could facilitate the clinical development of novel therapeutic and prognostic biomarkers for pGI-DLBCL.

## Supplementary Information

**Additional file 1: Table S1.** Clinicopathological information of 53 pGI-DLBCL patients.

**Additional file 2: Table S2.** Exonic mutation profile of 53 pGI-DLBCL patients.

**Additional file 3: Table S3.** KEGG enrichment results of recurrent driver genes in pGI-DLBCL.

**Additional file 4: Table S4. **Summary of the statistically significant correlations in the matrix.

**Additional file 5: Table S5.** Summary of IGLL5 and LRP1B mutations in pGI-DLBCL.

## Data Availability

The data that support the findings of this study are available from the corresponding authors upon reasonable request.

## Abbreviations

ABC: Activated B-cell-like
CLL: Chronic lymphocytic leukaemia
COSMIC: Catalog of somatic mutations in cancer
DAVID: Database for annotation, visualization and integrated discovery
DLBCL: Diffuse large B-cell lymphoma;
ECOG: Eastern cooperative oncology group
GCB: Germinal center B-cell-like
HBV: Hepatitis B virus
Hp: Helicobacter pylori
InDel: Insertion or deletion
IPI: International prognostic index
KEGG: Kyoto encyclopedia of genes and genomes
LM-PCR: Ligation-mediated PCR
NHL: Non-Hodgkin lymphoma
OS: Overall survival
PBMCs: Peripheral blood mononuclear cells
pGI-DLBCL: Primary gastrointestinal diffuse large B-cell lymphoma
R-CHOP: Rituximab plus cyclophosphomide, doxorubicin, vincristine and prednisone
SNV: Single nucleotide variant
TSG: Tumor suppressor gene
WES: Whole-exome sequencing
