# Supplementary material for: Whole-exome sequencing analysis identifies distinct mutational profile and novel prognostic biomarkers in primary gastrointestinal diffuse large B-cell lymphoma
Source: Exp Hematol Oncol. 2022 Oct 15;11:71. doi: 10.1186/s40164-022-00325-7 (PMC9569083; doi:10.1186/s40164-022-00325-7)
Supplement: Supplementary file 3 — Additional file 3: Table S3. KEGG enrichment results of recurrent driver genes in pGI-DLBCL. [file 40164_2022_325_MOESM3_ESM.docx]

**Supplementary Table 3.** KEGG enrichment results of recurrent driver genes in pGI-DLBCL.

| KEGG Term | Genes | *P* Value | Bonferroni |
| --- | --- | --- | --- |
| hsa05200:Pathways in cancer | RET, CSF1R, HSP90AB1, TCF7, PTEN, CXCR4, PIK3CD, CBLB, ADCY1, PIK3CB, FOXO1, IKBKB, MYC, EP300, CTNNA2, PDGFRA, MAP2K1, CREBBP, HSP90AA1, PRKCB, DCC, HGF, STAT3, BRAF, MTOR, BCR, NFKBIA, APC, MSH3, GNAQ, KIT, RARA, FAS, KRAS, TP53, FGFR2 | 5.04E-17 | 9.01E-15 |
| hsa05215:Prostate cancer | PDGFRA, MAP2K1, CREBBP, HSP90AA1, HSP90AB1, TCF7, PTEN, PIK3CD, BRAF, PIK3CB, FOXO1, MTOR, NFKBIA, IKBKB, EP300, KRAS, TP53, FGFR2 | 4.95E-14 | 8.86E-12 |
| hsa05221:Acute myeloid leukemia | MAP2K1, TCF7, STAT3, PIK3CD, BRAF, PIK3CB, MTOR, IKBKB, MYC, KIT, PIM1, RARA, KRAS | 8.90E-11 | 1.59E-08 |
| **hsa04919:Thyroid hormone signaling pathway** | **MAP2K1, CREBBP, NOTCH1, PRKCB, PIK3CD, PIK3CB, FOXO1, MTOR, ACTB, ACTG1, NCOR1, SIN3A, MYC, EP300, KRAS, TP53** | **5.94E-10** | **1.06E-07** |
| **hsa05230:Central carbon metabolism in cancer** | **RET, PDGFRA, MAP2K1, MYC, KIT, PTEN, PIK3CD, KRAS, PIK3CB, TP53, FGFR2, MTOR** | **6.82E-09** | **1.22E-06** |
| hsa05213:Endometrial cancer | MAP2K1, APC, MYC, TCF7, PTEN, PIK3CD, BRAF, KRAS, CTNNA2, PIK3CB, TP53 | 1.10E-08 | 1.96E-06 |
| **hsa05161:Hepatitis B** | **MAP2K1, CREBBP, DDX3X, PRKCB, STAT3, PTEN, NFATC2, PIK3CD, PIK3CB, NFKBIA, IKBKB, MYC, FAS, EP300, KRAS, TP53** | **1.55E-08** | **2.77E-06** |
| **hsa04662:B cell receptor signaling pathway** | **NFKBIA, CD79B, IKBKB, MAP2K1, NFATC2, PIK3CD, PTPN6, KRAS, PIK3CB, NFKBIE, CARD11, MALT1** | **1.56E-08** | **2.79E-06** |
| **hsa04068:FoxO signaling pathway** | **MAP2K1, CREBBP, STAT3, PTEN, PIK3CD, BRAF, IRS2, PIK3CB, FOXO1, IKBKB, BCL6, EP300, KRAS, SGK1, RAG1** | **4.28E-08** | **7.65E-06** |
| hsa05210:Colorectal cancer | MAP2K1, APC, MSH3, DCC, MYC, TCF7, PIK3CD, BRAF, KRAS, PIK3CB, TP53 | 6.48E-08 | 1.16E-05 |
| hsa05206:MicroRNAs in cancer | PDGFRA, MAP2K1, CREBBP, NOTCH1, ABCB1, PRKCB, STAT3, PTEN, IRS2, MTOR, IKBKB, ZEB2, SOCS1, APC, MYC, PIM1, EP300, KRAS, TP53, TP63 | 2.38E-07 | 4.26E-05 |
| hsa05220:Chronic myeloid leukemia | BCR, NFKBIA, IKBKB, MAP2K1, MYC, PIK3CD, BRAF, CBLB, KRAS, PIK3CB, TP53 | 2.80E-07 | 5.02E-05 |
| hsa04015:Rap1 signaling pathway | PDGFRA, CSF1R, MAP2K1, PRKCB, HGF, PIK3CD, BRAF, ADCY1, PIK3CB, ACTB, ACTG1, GRIN2A, GNAQ, KIT, KRAS, FGFR4, FGFR2 | 3.76E-07 | 6.73E-05 |
| hsa04660:T cell receptor signaling pathway | NFKBIA, IKBKB, MAP2K1, PTPRC, NFATC2, PIK3CD, PTPN6, KRAS, PIK3CB, NFKBIE, CARD11, MALT1 | 7.80E-07 | 1.40E-04 |
| hsa04151:PI3K-Akt signaling pathway | PDGFRA, CSF1R, MAP2K1, HSP90AA1, HSP90AB1, HGF, PTEN, PIK3CD, PIK3CB, MTOR, IKBKB, CCND3, COL3A1, TCL1A, MYC, KIT, KRAS, SGK1, FGFR4, TP53, FGFR2 | 9.90E-07 | 1.77E-04 |
| hsa05205:Proteoglycans in cancer | MAP2K1, PRKCB, HGF, STAT3, PIK3CD, BRAF, CBLB, PIK3CB, MTOR, ACTB, ACTG1, MYC, FAS, KRAS, PTPN6, TP53 | 1.10E-06 | 1.97E-04 |
| hsa05214:Glioma | PDGFRA, MAP2K1, PRKCB, PTEN, PIK3CD, BRAF, KRAS, PIK3CB, TP53, MTOR | 1.17E-06 | 2.09E-04 |
| hsa05166:HTLV-I infection | PDGFRA, CREBBP, TRRAP, HLA-B, NFATC2, PIK3CD, HLA-A, ADCY1, PIK3CB, NFKBIA, IKBKB, CCND3, APC, MYC, EP300, KRAS, TP53 | 4.72E-06 | 8.44E-04 |
| hsa05169:Epstein-Barr virus infection | NFKBIA, NCOR2, IKBKB, MYC, STAT3, HLA-B, TNFAIP3, PIK3CD, HLA-A, PIK3CB, NFKBIE, TP53 | 5.66E-06 | 1.01E-03 |
| hsa05216:Thyroid cancer | RET, MAP2K1, MYC, TCF7, BRAF, KRAS, TP53 | 6.99E-06 | 1.25E-03 |
| hsa04720:Long-term potentiation | MAP2K1, GRIN2A, CREBBP, PRKCB, GNAQ, EP300, BRAF, KRAS, ADCY1 | 1.30E-05 | 2.33E-03 |
| hsa04012:ErbB signaling pathway | MAP2K1, PRKCB, MYC, PIK3CD, BRAF, CBLB, KRAS, NRG1, PIK3CB, MTOR | 1.38E-05 | 2.46E-03 |
| hsa04310:Wnt signaling pathway | SFRP4, CCND3, CREBBP, APC, TBL1XR1, PRKCB, CHD8, MYC, TCF7, EP300, NFATC2, TP53 | 1.85E-05 | 3.31E-03 |
| hsa05218:Melanoma | PDGFRA, MAP2K1, HGF, PTEN, PIK3CD, BRAF, KRAS, PIK3CB, TP53 | 2.25E-05 | 4.02E-03 |
| hsa04722:Neurotrophin signaling pathway | NFKBIA, IKBKB, NTRK2, MAP2K1, PRKCD, PIK3CD, BRAF, KRAS, PIK3CB, NFKBIE, TP53 | 3.08E-05 | 5.50E-03 |
| hsa05100:Bacterial invasion of epithelial cells | CLTCL1, CLTC, PIK3CD, CBLB, CTNNA2, PIK3CB, ACTB, ACTG1, DNM2 | 4.50E-05 | 8.02E-03 |
| hsa04931:Insulin resistance | NFKBIA, IKBKB, STAT3, PRKCD, PTEN, PIK3CD, IRS2, PIK3CB, FOXO1, MTOR | 7.81E-05 | 1.39E-02 |
| hsa05212:Pancreatic cancer | IKBKB, MAP2K1, STAT3, PIK3CD, BRAF, KRAS, PIK3CB, TP53 | 1.00E-04 | 1.78E-02 |
| hsa04014:Ras signaling pathway | PDGFRA, CSF1R, MAP2K1, PRKCB, HGF, PIK3CD, PIK3CB, IKBKB, GRIN2A, RASA1, KIT, KRAS, FGFR4, FGFR2 | 1.01E-04 | 1.79E-02 |
| hsa04910:Insulin signaling pathway | IKBKB, MAP2K1, SOCS1, PIK3CD, BRAF, CBLB, IRS2, KRAS, PIK3CB, FOXO1, MTOR | 1.02E-04 | 1.80E-02 |
| hsa05211:Renal cell carcinoma | MAP2K1, CREBBP, HGF, EP300, PIK3CD, BRAF, KRAS, PIK3CB | 1.11E-04 | 1.96E-02 |
| hsa04550:Signaling pathways regulating pluripotency of stem cells | MAP2K1, ZFHX3, APC, MYC, STAT3, ID3, PIK3CD, KRAS, PIK3CB, FGFR4, FGFR2 | 1.15E-04 | 2.03E-02 |
| hsa04930:Type II diabetes mellitus | IKBKB, SOCS1, PRKCD, PIK3CD, IRS2, PIK3CB, MTOR | 1.39E-04 | 2.46E-02 |
| hsa05164:Influenza A | NFKBIA, IKBKB, MAP2K1, CIITA, CREBBP, PRKCB, EP300, FAS, PIK3CD, PIK3CB, ACTB, ACTG1 | 1.55E-04 | 2.74E-02 |
| hsa05203:Viral carcinogenesis | CREBBP, DDX3X, STAT3, HLA-B, PIK3CD, CHD4, HLA-A, PIK3CB, NFKBIA, CCND3, EP300, KRAS, TP53 | 1.61E-04 | 2.83E-02 |
| hsa04520:Adherens junction | CREBBP, PTPRB, TCF7, EP300, PTPN6, CTNNA2, ACTB, ACTG1 | 1.76E-04 | 3.11E-02 |
| hsa04915:Estrogen signaling pathway | MAP2K1, HSP90AA1, HSP90AB1, GNAQ, PRKCD, PIK3CD, KRAS, ADCY1, PIK3CB | 2.45E-04 | 4.29E-02 |
| hsa04916:Melanogenesis | MAP2K1, CREBBP, PRKCB, GNAQ, TCF7, KIT, EP300, KRAS, ADCY1 | 2.63E-04 | 4.59E-02 |
