# Supplementary material for: Whole-exome sequencing analysis identifies distinct mutational profile and novel prognostic biomarkers in primary gastrointestinal diffuse large B-cell lymphoma
Source: Exp Hematol Oncol. 2022 Oct 15;11:71. doi: 10.1186/s40164-022-00325-7 (PMC9569083; doi:10.1186/s40164-022-00325-7)
Supplement: Supplementary file 4 — Additional file 4: Table S4. Summary of the statistically significant correlations in the matrix. [file 40164_2022_325_MOESM4_ESM.docx]

**Supplementary Table 4.** Summary of the statistically significant correlations in the matrix.

| **Parameter 1** | **Parameter 2** | **r** | **P** |
| --- | --- | --- | --- |
| Age | ECOG_PS | 0.423 | 0.002 |
| HBsAg | HBsAb | -0.502 | 0.000 |
| HBsAg | HBeAg | 0.271 | 0.050 |
| HBsAg | HBeAb | 0.441 | 0.001 |
| HBsAg | HBcAb | 0.415 | 0.002 |
| HBeAb | HBcAb | 0.656 | 0.000 |
| Gender | LDH | 0.391 | 0.004 |
| Hp_Infection | LDH | 0.369 | 0.007 |
| Age | Hypoproteinemia | 0.319 | 0.020 |
| Hypoproteinemia | Anemia | 0.329 | 0.016 |
| B_Symptom | Lugano_Staging | 0.303 | 0.028 |
| LDH | Lugano_Staging | 0.444 | 0.001 |
| Gender | IPI | 0.301 | 0.028 |
| Age | IPI | 0.569 | 0.000 |
| ECOG_PS | IPI | 0.452 | 0.001 |
| B_Symptom | IPI | 0.360 | 0.008 |
| Hp_Infection | IPI | 0.378 | 0.005 |
| LDH | IPI | 0.658 | 0.000 |
| Hypoproteinemia | IPI | 0.280 | 0.042 |
| Lugano_Staging | IPI | 0.635 | 0.000 |
| HBsAg | TP53_Mutation | 0.324 | 0.018 |
| B_Symptom | PCLO_Mutation | 0.289 | 0.036 |
| LDH | PCLO_Mutation | -0.276 | 0.045 |
| HBsAb | HIST1H1E_Mutation | 0.335 | 0.014 |
| BTG2_Mutation | IGHM_Mutation | 0.401 | 0.003 |
| P2RY8_Mutation | IGHM_Mutation | -0.307 | 0.026 |
| HBsAb | CSMD3_Mutation | 0.298 | 0.030 |
| PCLO_Mutation | CSMD3_Mutation | 0.430 | 0.001 |
| Primary_Site | MUC16_Mutation | -0.308 | 0.025 |
| PCLO_Mutation | MUC16_Mutation | 0.430 | 0.001 |
| CSMD3_Mutation | MUC16_Mutation | 0.384 | 0.005 |
| HBeAg | RYR2_Mutation | 0.288 | 0.037 |
| IGLL5_Mutation | RYR2_Mutation | 0.317 | 0.021 |
| LDH | DUSP2_Mutation | -0.333 | 0.015 |
| BTG2_Mutation | DUSP2_Mutation | 0.274 | 0.047 |
| P2RY8_Mutation | DUSP2_Mutation | 0.299 | 0.030 |
| Age | FAT4_Mutation | -0.283 | 0.040 |
| HBeAg | FAT4_Mutation | 0.307 | 0.026 |
| Lugano_Staging | FAT4_Mutation | -0.272 | 0.048 |
| P2RY8_Mutation | FAT4_Mutation | 0.299 | 0.030 |
| BTG2_Mutation | IGHJ6_Mutation | 0.274 | 0.047 |
| Hans_Algorithm | CARD11_Mutation | 0.324 | 0.018 |
| HIST1H1E_Mutation | HIST1H1C_Mutation | 0.304 | 0.027 |
| HBsAg | LRP1B_Mutation | 0.304 | 0.027 |
| HBeAg | LRP1B_Mutation | 0.329 | 0.016 |
| TP53_Mutation | MYC_Mutation | 0.287 | 0.037 |
| HIST1H1C_Mutation | SI_Mutation | 0.411 | 0.002 |
| MYC_Mutation | SI_Mutation | 0.411 | 0.002 |
| HBeAb | B2M_Mutation | 0.368 | 0.007 |
| P2RY8_Mutation | B2M_Mutation | 0.272 | 0.049 |
| FAT4_Mutation | B2M_Mutation | 0.417 | 0.002 |
| HIST1H1C_Mutation | B2M_Mutation | 0.303 | 0.028 |
| HIST1H1C_Mutation | BTG1_Mutation | 0.303 | 0.028 |
| B2M_Mutation | BTG1_Mutation | 0.342 | 0.012 |
| RYR2_Mutation | CSMD1_Mutation | 0.382 | 0.005 |
| CARD11_Mutation | CSMD1_Mutation | 0.458 | 0.001 |
| HIST1H1E_Mutation | EBF1_Mutation | 0.350 | 0.010 |
| HIST1H1C_Mutation | EBF1_Mutation | 0.303 | 0.028 |
| SI_Mutation | EYS_Mutation | 0.303 | 0.028 |
| B2M_Mutation | EYS_Mutation | 0.506 | 0.000 |
| Age | FOXO1_Mutation | -0.297 | 0.031 |
| HBsAb | FOXO1_Mutation | 0.286 | 0.038 |
| Anemia | FOXO1_Mutation | -0.355 | 0.009 |
| IGLL5_Mutation | FOXO1_Mutation | 0.301 | 0.028 |
| MYC_Mutation | FOXO1_Mutation | 0.458 | 0.001 |
| NBPF1_Mutation | FOXO1_Mutation | 0.303 | 0.028 |
| Primary_Site | GNAI2_Mutation | -0.291 | 0.035 |
| Hans_Algorithm | GNAI2_Mutation | -0.304 | 0.027 |
| Lugano_Staging | IGHA1_Mutation | -0.387 | 0.004 |
| CSMD3_Mutation | IGHA1_Mutation | 0.382 | 0.005 |
| SI_Mutation | IGHA1_Mutation | 0.303 | 0.028 |
| HBsAb | TET2_Mutation | 0.286 | 0.038 |
| NBPF1_Mutation | TET2_Mutation | 0.303 | 0.028 |
| FOXO1_Mutation | TET2_Mutation | 0.342 | 0.012 |
| IGHA1_Mutation | TET2_Mutation | 0.342 | 0.012 |
